# Supplementary material for: CapTrap-seq: a platform-agnostic and quantitative approach for high-fidelity full-length RNA sequencing
Source: Nat Commun. 2024 Jun 27;15:5278. doi: 10.1038/s41467-024-49523-3 (PMC11211341; doi:10.1038/s41467-024-49523-3)
Supplement: Supplementary file 1 — Supplementary Information [file 41467_2024_49523_MOESM1_ESM.pdf]

Supplementary Information

CapTrap-Seq: A platform-agnostic and quantitative approach for high-fidelity full-length RNA sequencing

Silvia Carbonell-Sala[1], Tamara Perteghella[1,2], Julien Lagarde[1,3], Hiromi Nishiyori[4], Emilio Palumbo[1], Carme Arnan[1], Hazuki Takahashi[4], Piero Carninci[4,5], Barbara Uszczynska-Ratajczak[1,6,#], Roderic Guigó[1,2,#]

1. Centre for Genomic Regulation (CRG), the Barcelona Institute of Science and Technology, Barcelona, Catalonia, Spain.
2. Universitat Pompeu Fabra, Barcelona, Catalonia, Spain
3. Flomics Biotech, SL, Carrer de Roc Boronat 31, 08005 Barcelona, Spain
4. Laboratory for Transcriptome Technology, RIKEN Center for Integrative Medical Sciences (IMS), Yokohama, Kanagawa, Japan
5. Human Technopole, Milan, Italy.
6. Institute of Bioorganic Chemistry, Polish Academy of Sciences, Poznan, Poland.

# Corresponding author

Correspondence should be addressed to R.G. (roderic.guigo@crg.cat) or B.U.R (barbara.uszczynska@gmail.com).

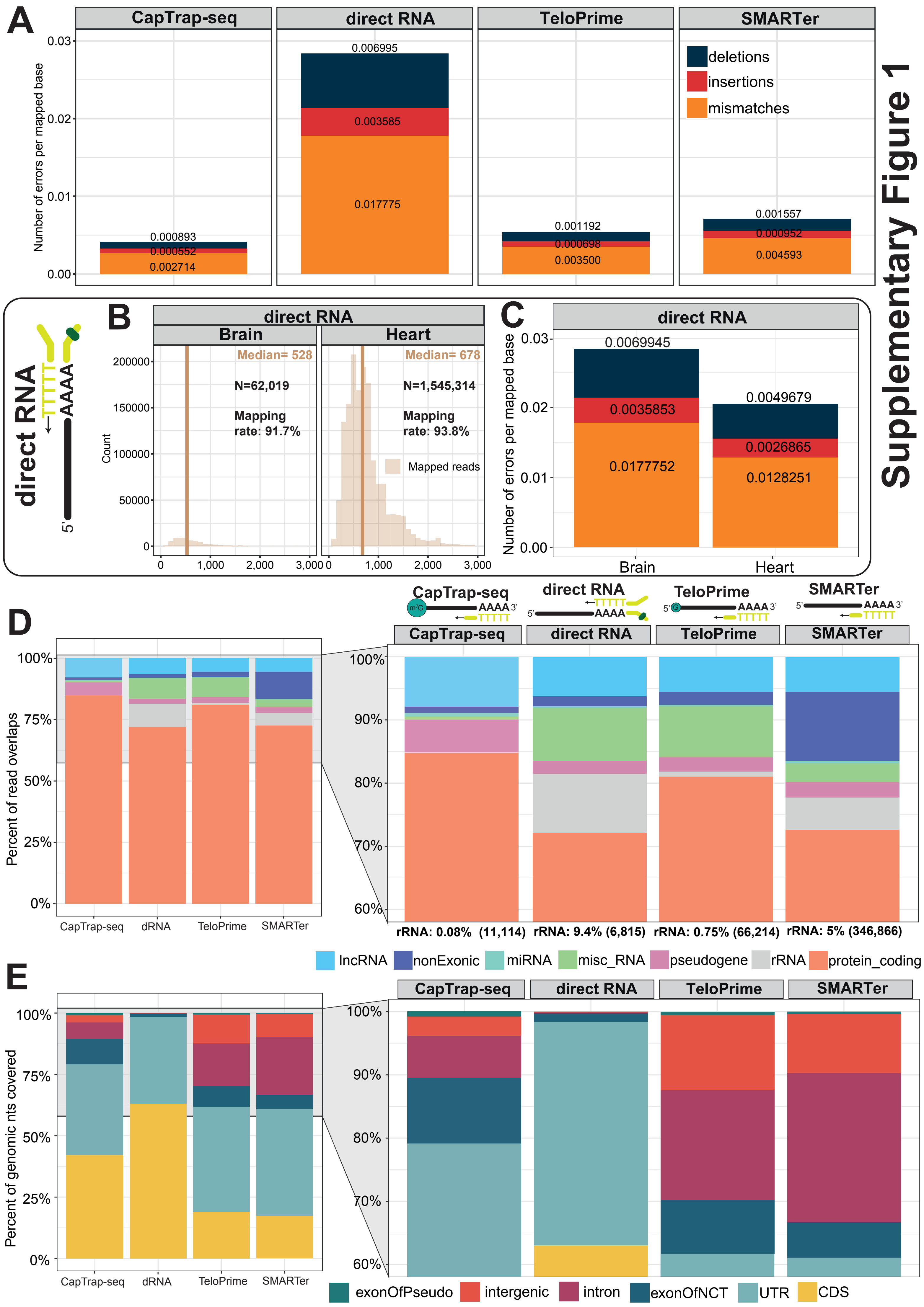

Supplementary Figure 1
A
CapTrap-seq
direct RNA
TeloPrime
SMARTer
Number of errors per mapped base
deletions
insertions
mismatches
0.000893
0.000552
0.002714
0.006995
0.003585
0.017775
0.001192
0.000698
0.003500
0.001557
0.000952
0.004593
direct RNA
B
direct RNA
Brain
Heart
Median= 528
Median= 678
N=62,019
N=1,545,314
Mapping rate: 91.7%
Mapping rate: 93.8%
Mapped reads
Count
C
direct RNA
Number of errors per mapped base
0.0069945
0.0035853
0.0177752
0.0049679
0.0026865
0.0128251
Brain
Heart
D
Percent of read overlaps
CapTrap-seq
dRNA
TeloPrime
SMARTer
rRNA: 0.08% (11,114)
rRNA: 9.4% (6,815)
rRNA: 0.75% (66,214)
rRNA: 5% (346,866)
lncRNA
nonExonic
miRNA
misc_RNA
pseudogene
rRNA
protein_coding
E
Percent of genomic nts covered
exonOfPseudo
intergenic
intron
exonOfNCT
UTR
CDS

Supplementary Figure 1. Read Quality Control for the human brain sample sequenced on ONT using different library preparation methods. (A) The number of errors per mapped bases (error rate) across sequencing library preparation protocols (CapTrap-seq, directRNA, TeloPrime and SMARTer) in the human brain sample. Colors denote the type of error: deletions (black), insertions (red), mismatches (orange); (**B-C)** metrics for directRNA ONT sequencing in the human brain and heart samples as described in Figure 1D and S1A; **(D)** The GENCODE gene biotype detection by ONT sequencing of four tested libraries. The stacked bar plots report the percentage of raw read overlaps with annotated GENCODE genes (v24) of given biotype. Colors denote different GENCODE gene biotypes: protein-coding genes (orange), ribosomal RNAs (rRNAs, light gray), pseudogenes (pink), miscellaneous RNA (misc_RNA, green), miRNAs (olive green), non-exonic (dark blue), lncRNA (sky blue). The proportion of raw reads mapping to rRNA along with absolute raw read numbers in parenthesis is given below each bar plot; **(E)** The proportion of nucleotides covering different genomic partitions. Colors highlight different region types: exon of pseudogene (green), intron (pink), untranslated regions (UTR, blue), intergenic (orange), exon of noncoding transcript (navy) and coding sequence (CDS, yellow).

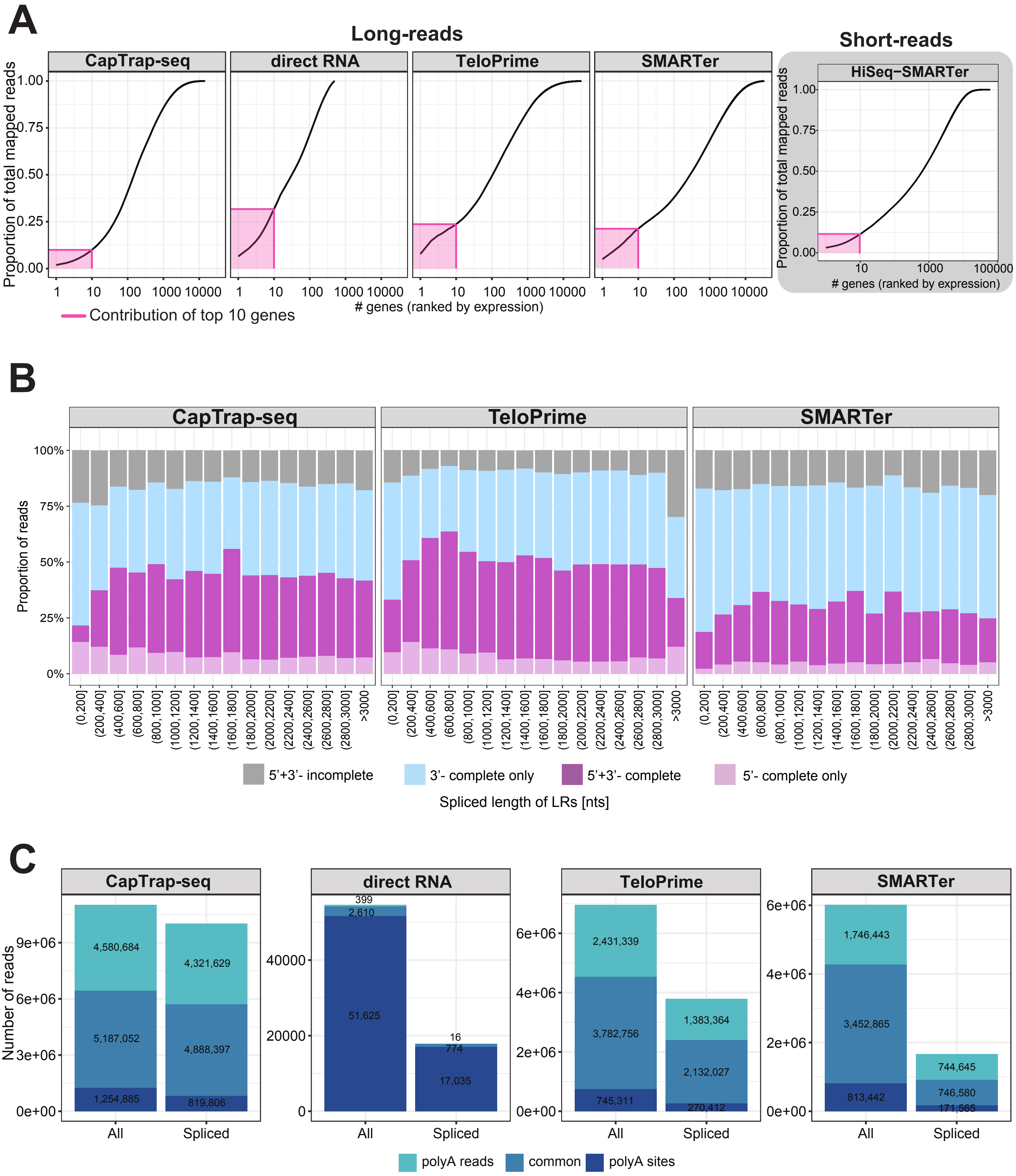

Supplementary Figure 2

Supplementary Figure 2. Mapping statistics for the human brain sample sequenced on ONT using different library preparation methods. (A) Cumulative transcript curve showing the proportion of mapped reads (y-axis) captured by the top N expressed genes (x-axis). The pink area represents the proportion of reads captured by the top ten gene expressors; **(B)** Length distribution of mapped long-read ONT reads for each protocol (CapTrap-seq, TeloPrime and SMARTer) binned by length. Colors highlights four different categories of long-read (LR) completeness. See Figure 1E for details; **(C)** Support of poly(A) reads by the presence of canonical polyadenylation motif[1] within a window of +/-50 bp[2]. Colors indicate various read types: solely supported by the polyA motif (dark blue), polyadenylated reads (green), or a combination of both (light blue).

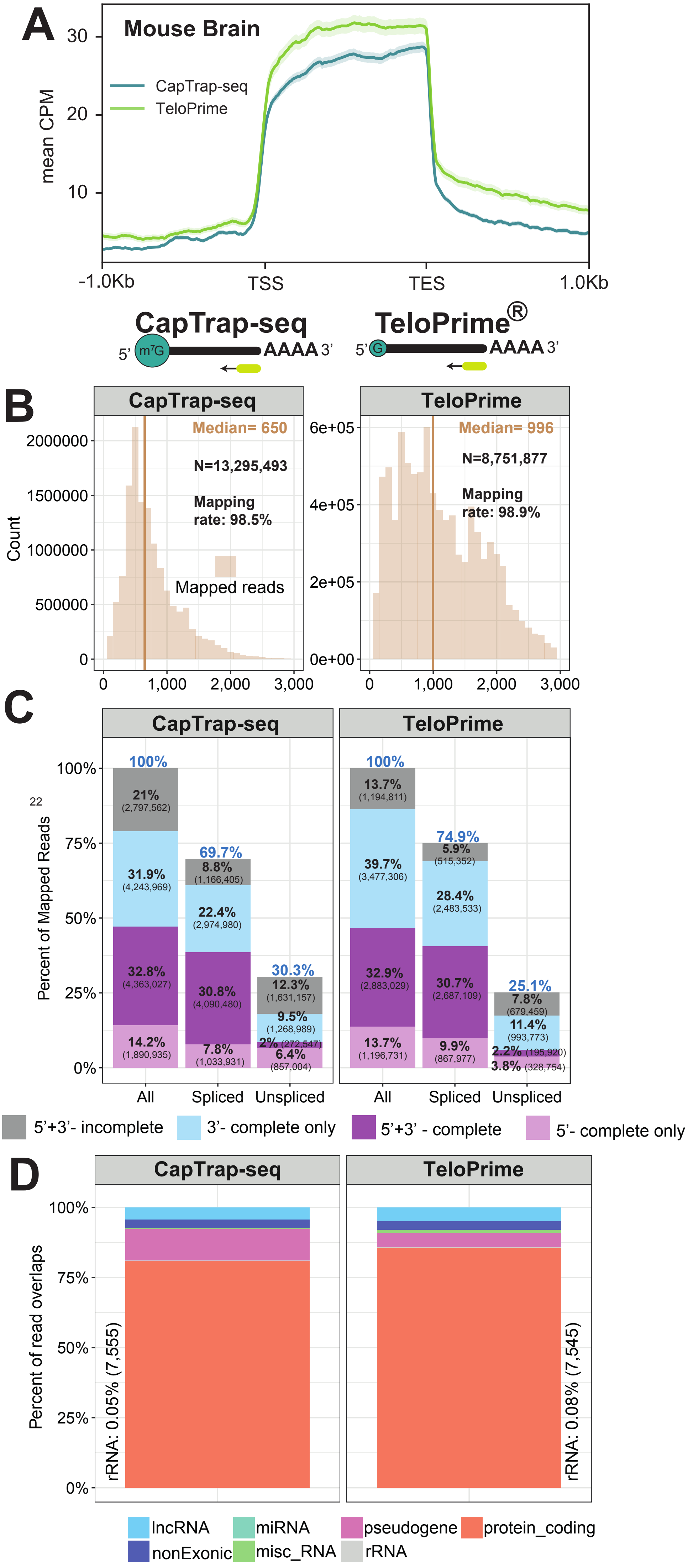

Supplementary Figure 3

Supplementary Figure 3. CapTrap-seq and Teloprime performance in mouse brain RNA sample sequenced on ONT. (A) Read aggregate deepTools2[3] profiles along the bodies of annotated GENCODE genes. The shaded areas represent the 95% confidence interval; **(B)** Length distribution for all mapped reads and **(C)** The proportion of reads with different types of termini support as described in Figure 1; **(D)** The GENCODE gene biotype detection by ONT sequencing as described in Figure S2A.

Supplementary Figure 4

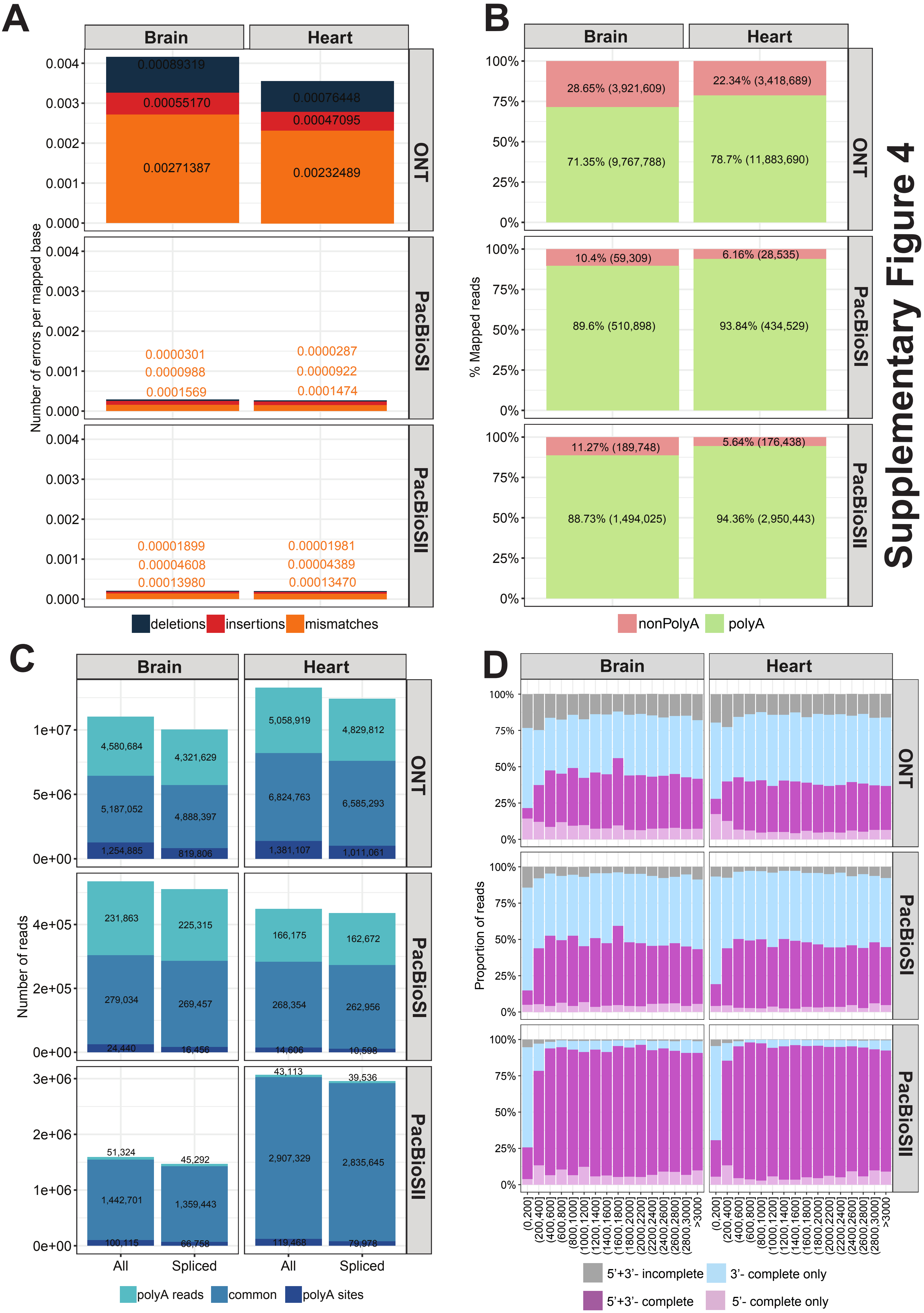

Supplementary Figure 4. Mapping statistics for CapTrap-seq across different sequencing platforms in human brain and heart. (A) The number of errors per mapped bases (error rate) for CapTrap-seq protocol sequenced using the ONT, PacBioSI and PacBioSII platforms. The color code as specified in Figure S1C; **(B)** Proportion of poly(A) (green) and non-poly(A) (red) ONT and PacBio (SI and SII) reads; **(C)** Support of poly(A) reads by the presence of canonical polyadenylation motif[1] within a window of +/-50 bp[2]. Legend as described in Figure S2C; **(D)** Detection of full-length reads in brain and heart samples, across the three sequencing technologies, binned by length. See Figure 1E for details.

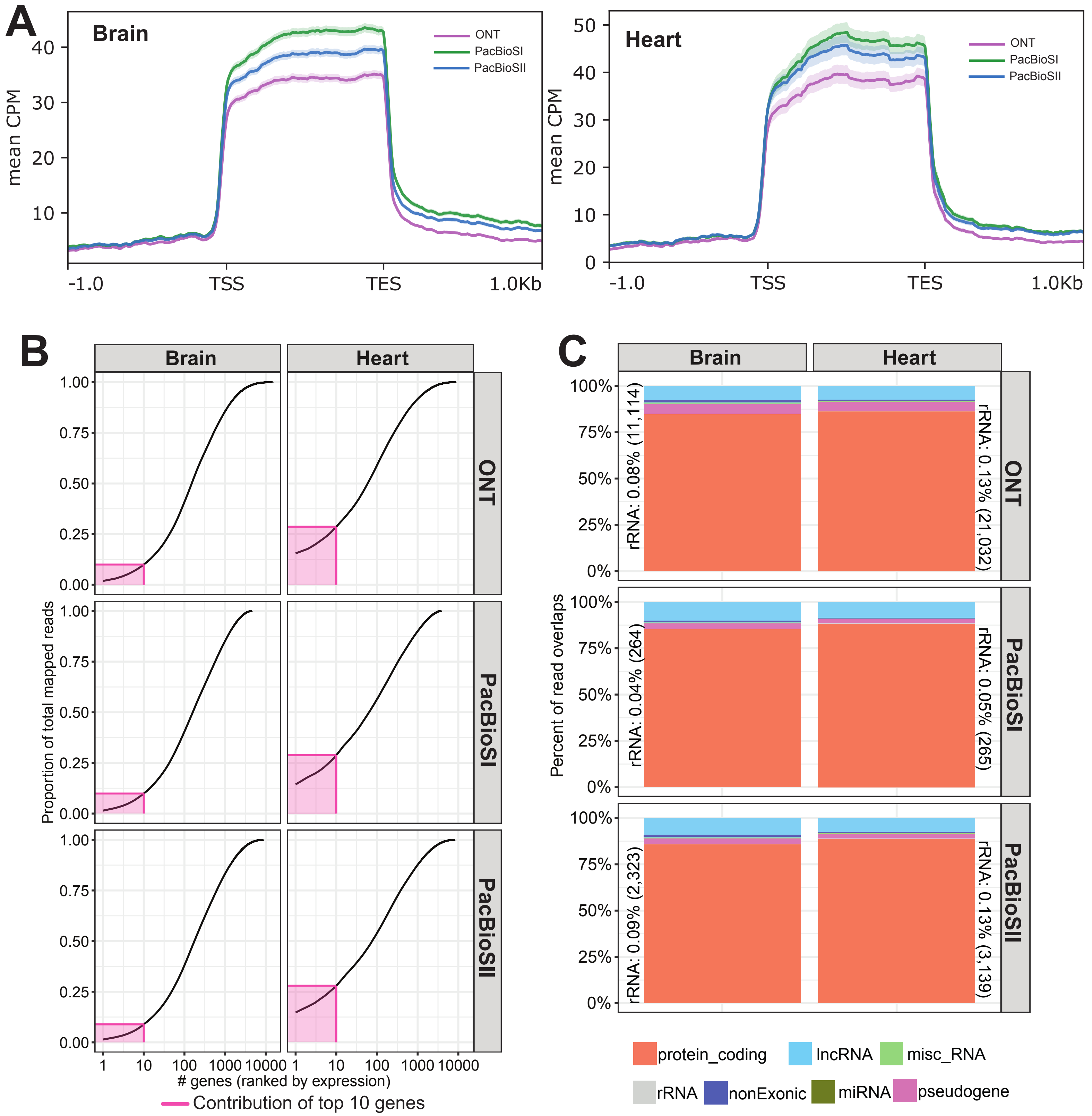

Supplementary Figure 5

Supplementary Figure 5. Mapping statistics for CapTrap-seq across different sequencing platforms in human brain and heart. (A) Read aggregate deepTools2[3] profiles along the bodies of annotated GENCODE genes for the CapTrap-seq combined with different long-read RNA sequencing platforms. The shaded regions represent the 95% confidence interval; **(B)** Cumulative transcript curve showing the proportion of mapped reads (y-axis) captured by the top N expressed genes (x-axis). The pink area represents the proportion of reads captured by the top ten gene expressors; **(C)** The GENCODE gene biotype detection for ONT and PacBio sequencing platforms. Biotype classes as described in Figure S2. The proportion of raw reads mapping to rRNA along with absolute raw read numbers in parenthesis is given on the outer side of each bar.

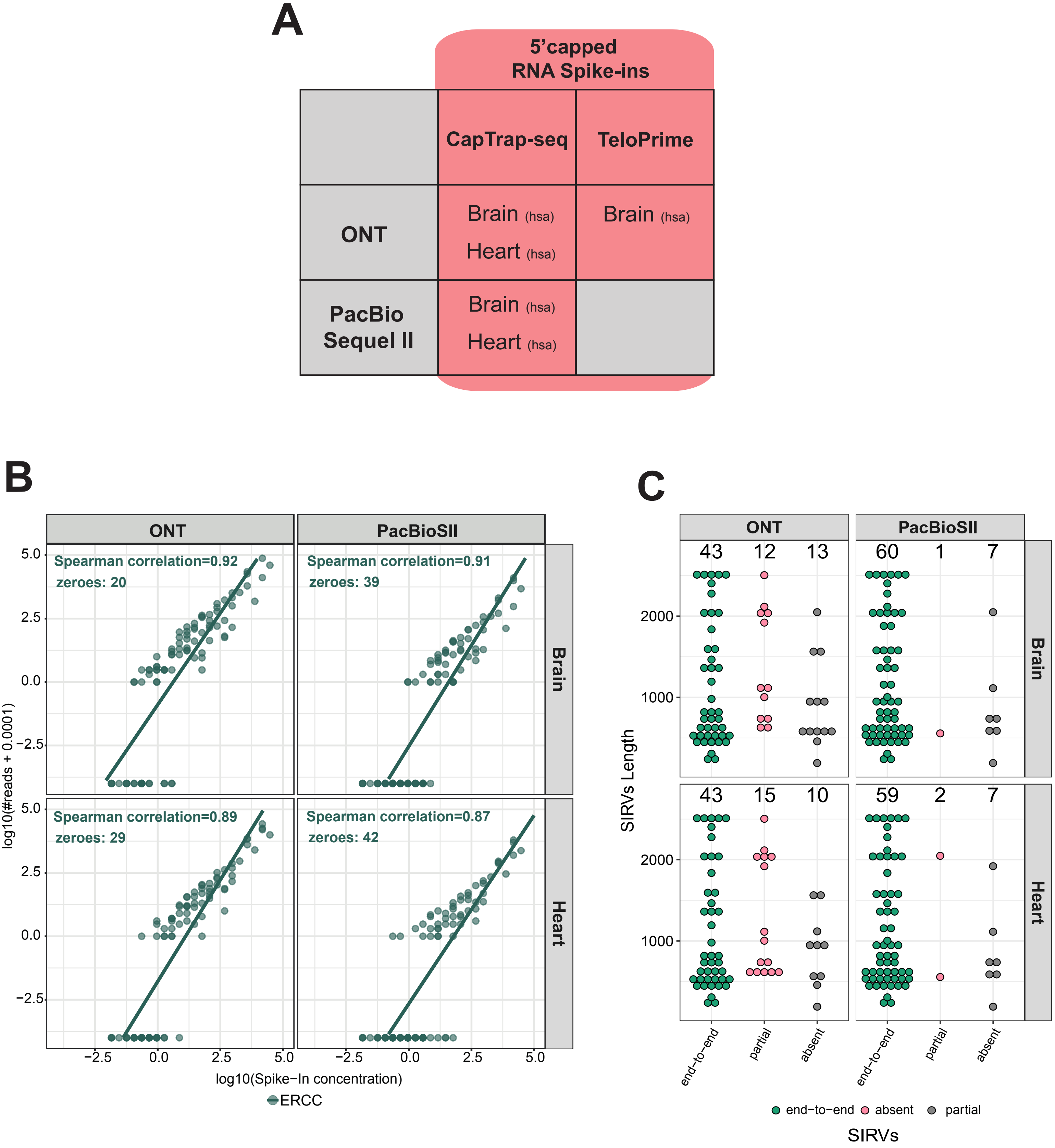

Supplementary Figure 6

Supplementary Figure 6. Capping of SIRVs and ERCC controls across tissues and platforms. (A) Experimental design for unbiased (92 ERCC spike-ins) CapTrap-seq experiments using the capped synthetic ERCC and SIRV controls. The coral-colored area indicates the samples available for this comparison; (**B**) Spearman correlation between input RNA concentration and raw read counts for ERCC spike-ins in the brain and heart samples. The individual data points correspond to 92 synthetic ERCC RNA sequences (for further details see Figure 4); **(C)** Detection of SIRVs as a function of length. Three main detection levels have been distinguished: end-to-end (green), partial (red), not detected/absent (gray). The black numbers displayed at the top indicate the total number of SIRVs for each detection level.

Supplementary Figure 7

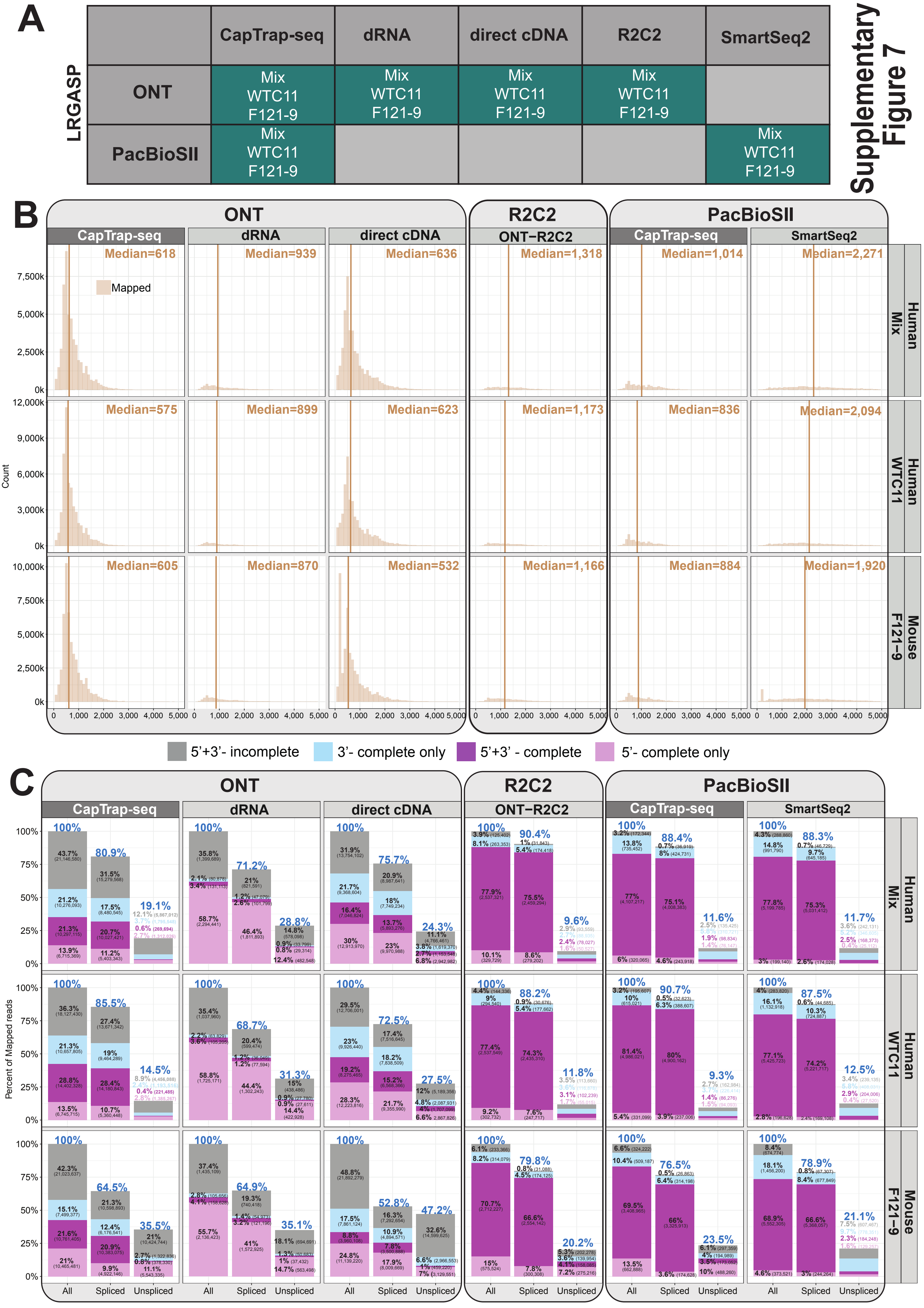

Supplementary Figure 7. Benchmark of CapTrap-seq using LRGASP samples. (A) Graphical summary of LRGASP samples and protocols selected for the CapTrap-seq benchmark. The areas highlighted in green indicate the samples available for this comparison; **(B)** Length distribution for all mapped reads and **(C)** The proportion of reads with different types of termini support as described in Figure 1; the gray shades highlight the platform or the sequencing methods in case of custom sequencing approaches, e.g. R2C2. To emphasize the compatibility of CapTrap-seq with both PacBioSII and ONT samples, it was shaded in a darker gray tone. For clarity, the triple replicates for each sample were combined together.

Supplementary References

1. Lopez, F., Granjeaud, S., Ara, T., Ghattas, B. & Gautheret, D. The disparate nature of ‘intergenic’ polyadenylation sites. *Rna* **12**, 1794–1801 (2006).
2. Uszczynska-Ratajczak, B., Lagarde, J., Frankish, A., Guigó, R. & Johnson, R. Towards a complete map of the human long non-coding RNA transcriptome. *Nat. Rev. Genet.* **19**, 535–548 (2018).
3. Ramírez, F. *et al.* deepTools2: a next generation web server for deep-sequencing data analysis. *Nucleic Acids Res.* **44**, W160–W165 (2016).
