# Supplementary material for: CapTrap-seq: a platform-agnostic and quantitative approach for high-fidelity full-length RNA sequencing
Source: Nat Commun. 2024 Jun 27;15:5278. doi: 10.1038/s41467-024-49523-3 (PMC11211341; doi:10.1038/s41467-024-49523-3)
Supplement: Supplementary file 3 — Reporting summary [file 41467_2024_49523_MOESM3_ESM.pdf]

Corresponding author(s): Barbara Uszczynska-Ratajczak & Roderic Guigo

Last updated by author(s): Jun 6, 2024

Reporting Summary

Nature Portfolio wishes to improve the reproducibility of the work that we publish. This form provides structure for consistency and transparency in reporting. For further information on Nature Portfolio policies, see our Editorial Policies and the Editorial Policy Checklist.

Statistics

For all statistical analyses, confirm that the following items are present in the figure legend, table legend, main text, or Methods section.

n/a	Confirmed	
☐	☒	The exact sample size (*n*) for each experimental group/condition, given as a discrete number and unit of measurement
☐	☒	A statement on whether measurements were taken from distinct samples or whether the same sample was measured repeatedly
☒	☐	The statistical test(s) used AND whether they are one- or two-sided<br>*Only common tests should be described solely by name; describe more complex techniques in the Methods section.*
☒	☐	A description of all covariates tested
☐	☒	A description of any assumptions or corrections, such as tests of normality and adjustment for multiple comparisons
☐	☒	A full description of the statistical parameters including central tendency (e.g. means) or other basic estimates (e.g. regression coefficient) AND variation (e.g. standard deviation) or associated estimates of uncertainty (e.g. confidence intervals)
☒	☐	For null hypothesis testing, the test statistic (e.g. *F*, *t*, *r*) with confidence intervals, effect sizes, degrees of freedom and *P* value noted<br>*Give P values as exact values whenever suitable.*
☒	☐	For Bayesian analysis, information on the choice of priors and Markov chain Monte Carlo settings
☒	☐	For hierarchical and complex designs, identification of the appropriate level for tests and full reporting of outcomes
☐	☒	Estimates of effect sizes (e.g. Cohen's *d*, Pearson's *r*), indicating how they were calculated

Our web collection on statistics for biologists contains articles on many of the points above.

Software and code

Policy information about availability of computer code

Data collection	No software was used.
Data analysis	1. Guppy v6 basecaller to get the fastq data for the samples sequenced using the Oxford Nanopore Technology (ONT) MinION platform.<br><br>2. tmerge<br>tmerge compares transcript structures (or read-to-genome alignments) present in the input and attempts to reduce transcript redundancy, i.e., merge compatible input transcripts into non-redundant transcript models. The program treats spliced and monoexonic reads separately (i.e., those are never merged together).<br>Code: https://github.com/guigolab/tmerge<br><br>3. samToPolyA<br>A utility to detect poly-adenylated sequencing reads, call on-genome polyA sites and infer the reads' strand based on reads-to-genome alignments in SAM format.<br>Code: https://github.com/julienlag/samToPolyA<br><br>4. cutom code<br>All custom code used for processing the raw data has been released via a dedicated GitHub repository. Each figure, both main and supplementary, is presented in its own separate folder, accompanied by a clear and self-explanatory README file.<br>Code: https://github.com/TamaraPerteghella/CapTrap-seq_benchmark_analysis

For manuscripts utilizing custom algorithms or software that are central to the research but not yet described in published literature, software must be made available to editors and reviewers. We strongly encourage code deposition in a community repository (e.g. GitHub). See the Nature Portfolio guidelines for submitting code & software for further information.

Data

Policy information about availability of data

All manuscripts must include a data availability statement. This statement should provide the following information, where applicable:

- Accession codes, unique identifiers, or web links for publicly available datasets
- A description of any restrictions on data availability
- For clinical datasets or third party data, please ensure that the statement adheres to our policy

Sequence data, including raw long-read PacBio, ONT and short-read Illumina RNA-seq data have been deposited in the ArrayExpress repository under accession number E-MTAB-13063 https://www.ebi.ac.uk/biostudies/arrayexpress/studies/E-MTAB-13063. The LRGASP data and documentation can be found at https://www.gencodegenes.org/pages/LRGASP/. Source data are provided with this paper and can be accessed at https://github.com/TamaraPerteghella/CapTrap-seq_benchmark_analysis.

Research involving human participants, their data, or biological material

Policy information about studies with human participants or human data. See also policy information about sex, gender (identity/presentation), and sexual orientation and race, ethnicity and racism.

Reporting on sex and gender	Although we are utilizing human and mouse samples, reporting on sex and gender is not applicable to our study as the research question and methodology do not involve investigating or analyzing sex or gender-related factors.
Reporting on race, ethnicity, or other socially relevant groupings	Given the nature and focus of our study, race, ethnicity, or other socially relevant groupings were not applicable, as our research primarily centered on a specific biological mechanism rather than social or demographic factors.
Population characteristics	Population characteristics are not applicable to our study as our research focuses on a specific subset. The human samples are Total RNA from commercially available primary tissues of the brain and heart. The references and lots used are as follows:<br>1) Total RNA from adult brain (Ambion - catalog number AM7962; lot numbers 1887911 and 1997739, Thermo Fisher).<br>2) Total RNA from adult heart (Ambion - catalog number AM7966; lot numbers 1866106 and 1906770, Thermo Fisher).<br><br>The total RNA extracted from the brain of adult C57BL/6 mice was sourced from the CRG-PRBB animal facility, utilizing flash-frozen tissue samples.
Recruitment	Recruitment was not applicable to our study as the data or samples used were obtained from commercial sources, as mentioned before.
Ethics oversight	Ethics oversight was not applicable to our study as the data or samples used were obtained from commercial sources, and the study did not involve direct involvement or interaction with human participants, thereby exempting it from institutional ethics review.

Note that full information on the approval of the study protocol must also be provided in the manuscript.

Field-specific reporting

Please select the one below that is the best fit for your research. If you are not sure, read the appropriate sections before making your selection.

☒ Life sciences ☐ Behavioural & social sciences ☐ Ecological, evolutionary & environmental sciences

For a reference copy of the document with all sections, see nature.com/documents/nr-reporting-summary-flat.pdf

Life sciences study design

All studies must disclose on these points even when the disclosure is negative.

Sample size	Sample size is not applicable to this study because it is a pilot study. Pilot studies are small-scale preliminary investigations conducted before a larger study. Their primary purpose is to test the feasibility of the research design, assess the appropriateness of data collection methods, or estimate the variability of outcomes. Sample size determination for pilot studies is often based on practical considerations rather than formal statistical calculations.
Data exclusions	Data exclusions were not applicable to our study as the dataset used was complete and did not contain any missing or excluded data points, ensuring that the entire dataset was utilized for analysis.
Replication	Replication was performed in our study using replicates to ensure consistency and reliability of the obtained results.
Randomization	Randomization is not relevant for our pilot study as the primary focus is to assess the feasibility of the research design, evaluate data collection methods, and explore potential outcomes. The aim of the pilot study is to gather initial insights rather than establishing statistical significance or generalizability, thus negating the need for randomization in participant assignment or intervention allocation.

Blinding	Blinding is not applicable to this study, as this is a general methods paper aiming to benchmark CapTrap-seq approach with other widely used library preparation methods and asses its performance using different platforms.

Reporting for specific materials, systems and methods

We require information from authors about some types of materials, experimental systems and methods used in many studies. Here, indicate whether each material, system or method listed is relevant to your study. If you are not sure if a list item applies to your research, read the appropriate section before selecting a response.

Materials & experimental systems

n/a	Involved in the study	
☒	☐	Antibodies
☒	☐	Eukaryotic cell lines
☒	☐	Palaeontology and archaeology
☒	☐	Animals and other organisms
☒	☐	Clinical data
☒	☐	Dual use research of concern
☒	☐	Plants

Methods

n/a	Involved in the study	
☒	☐	ChIP-seq
☒	☐	Flow cytometry
☒	☐	MRI-based neuroimaging

Plants

Seed stocks	N/A
Novel plant genotypes	N/A
Authentication	N/A
